# Beyond the sella: Expanded endoscopic endonasal approaches for pituitary tumors

**DOI:** 10.1093/noajnl/vdae086

**Published:** 2025-01-02

**Authors:** Erion Junior de Andrade, Saira Alli, Raj Sindwani, Varun R Kshettry, Pablo F Recinos

**Affiliations:** Department of Neurological Surgery, Cleveland Clinic Lerner College of Medicine of Case Western Reserve University, Cleveland, Ohio, USA; Section of Skull Base Surgery, Rose Ella Burkhardt Tumor & Neuro-Oncology Center, Neurological Institute, Cleveland Clinic, Cleveland, Ohio, USA; Department of Neurological Surgery, Cleveland Clinic Lerner College of Medicine of Case Western Reserve University, Cleveland, Ohio, USA; Section of Skull Base Surgery, Rose Ella Burkhardt Tumor & Neuro-Oncology Center, Neurological Institute, Cleveland Clinic, Cleveland, Ohio, USA; Department of Neurological Surgery, Cleveland Clinic Lerner College of Medicine of Case Western Reserve University, Cleveland, Ohio, USA; Section of Skull Base Surgery, Rose Ella Burkhardt Tumor & Neuro-Oncology Center, Neurological Institute, Cleveland Clinic, Cleveland, Ohio, USA; Department of Otolaryngology-Head & Neck Surgery, Cleveland Clinic Lerner College of Medicine of Case Western Reserve University, Cleveland, Ohio, USA; Department of Neurological Surgery, Cleveland Clinic Lerner College of Medicine of Case Western Reserve University, Cleveland, Ohio, USA; Section of Skull Base Surgery, Rose Ella Burkhardt Tumor & Neuro-Oncology Center, Neurological Institute, Cleveland Clinic, Cleveland, Ohio, USA; Department of Otolaryngology-Head & Neck Surgery, Cleveland Clinic Lerner College of Medicine of Case Western Reserve University, Cleveland, Ohio, USA; Department of Neurological Surgery, Cleveland Clinic Lerner College of Medicine of Case Western Reserve University, Cleveland, Ohio, USA; Section of Skull Base Surgery, Rose Ella Burkhardt Tumor & Neuro-Oncology Center, Neurological Institute, Cleveland Clinic, Cleveland, Ohio, USA; Department of Otolaryngology-Head & Neck Surgery, Cleveland Clinic Lerner College of Medicine of Case Western Reserve University, Cleveland, Ohio, USA

## Abstract

Expanded endoscopic endonasal approaches (EEAs) have significantly advanced the surgical management of invasive pituitary tumors that extend beyond the sella turcica. They are particularly important in functioning tumors to achieve biochemical remission. In this article, we review the classification and application of expanded EEAs in addressing tumors invading the anterior skull base, suprasellar cisterns, clivus, and cavernous sinus. The anatomical basis, techniques, and indications for the endoscopic endonasal transtuberculum–transplanum, transclival, and transcavernous approaches, as well as the resection of the medial wall of the cavernous sinus, are discussed. The outcomes of these approaches are reviewed and our surgical strategy for these tumors is presented. Despite advances in technology and our understanding of the parasellar anatomy, we emphasize the importance of a multidisciplinary team and graded experiential learning for surgeons to minimize the complication rates associated with these technically advanced approaches.

 Endoscopic endonasal approaches (EEAs) to the skull base have significantly improved the management of pituitary tumors (PTs) allowing better exposure, visualization, and access to previously inaccessible areas.^1,2^ Although many PTs remain confined to the sella, a substantial number is infiltrative, invading the cavernous sinus (CS), anterior skull base, suprasellar cistern, or clivus. For invasive tumors, the standard endoscopic transsphenoidal approach is often insufficient for adequate exposure and complete tumor removal.^3–5^

The primary goal of surgical intervention for PTs is maximum safe resection while preserving the pituitary gland and surrounding neurovascular structures.^3,6^ This is especially important for functioning PTs, where the goal is complete tumor resection to achieve biochemical remission.^7–10^ In these cases, the invasiveness of the tumor is correlated with several clinical outcomes, including extent of surgical resection, risk of intraoperative complications, and risk of tumor recurrence.^11^ For this reason, the knowledge and adoption of expanded EEAs may allow for the resection of suprasellar and parasellar tumors that would otherwise require a transcranial approach.^12^

Classification of expanded EEAs can be separated into sagittal plane and coronal plane approaches. The ideal approach is selected by the location of the tumor invasion and its relationship with surrounding neurovascular structures.^13,14^ In the sagittal plane, endoscopic approaches commonly used for PTs can be classified as transtuberculum–transplanum (TT-TP) or transclival approaches.^15^ In the coronal plane, endoscopic approaches to the CS pose more of a challenge due to the critical neurovascular contents. Therefore, understanding the surgical anatomy and applications of the different surgical approaches is paramount. In this article, we review expanded EEAs for PTs.

## Extrasellar Growth of Pituitary Tumors

The growth rate of PTs is influenced by various patient- and tumor-specific characteristics including age, sex, hormone-secreting subtype, and immunohistochemical profile.^16^ PTs have 2 primary patterns of invasion: expansive growth, when the tumor grows in a manner that exerts pressure on and displaces the surrounding tissues without infiltration, and infiltrative growth which occurs when the tumor breaches the sellar walls extending through the dura mater into bone or the intradural space.^17,18^

The pituitary gland is located within the sella turcica of the sphenoid bone and is enclosed by dura mater. Its anterior, inferior, and posterior surfaces are shielded by a dual-layered dura mater and sellar bone, forming a robust barrier against tumor expansion in these planes. In contrast, the lateral and superior surfaces of the gland are enclosed by a single layer of dura mater.^19^ Furthermore, the lateral surfaces constitute the medial walls of the CS. In addition, veins that drain the gland into the CS traverse the medial wall, and inadvertently create susceptible points along these walls.^19–21^ The superior surface of the gland is covered by the diaphragma sellae that is composed of 2 dural layers. An opening for the infundibulum is present in the center, which has a variable size ranging from 2.8 to 14.1 mm.^21,22^

**Figure 1.**
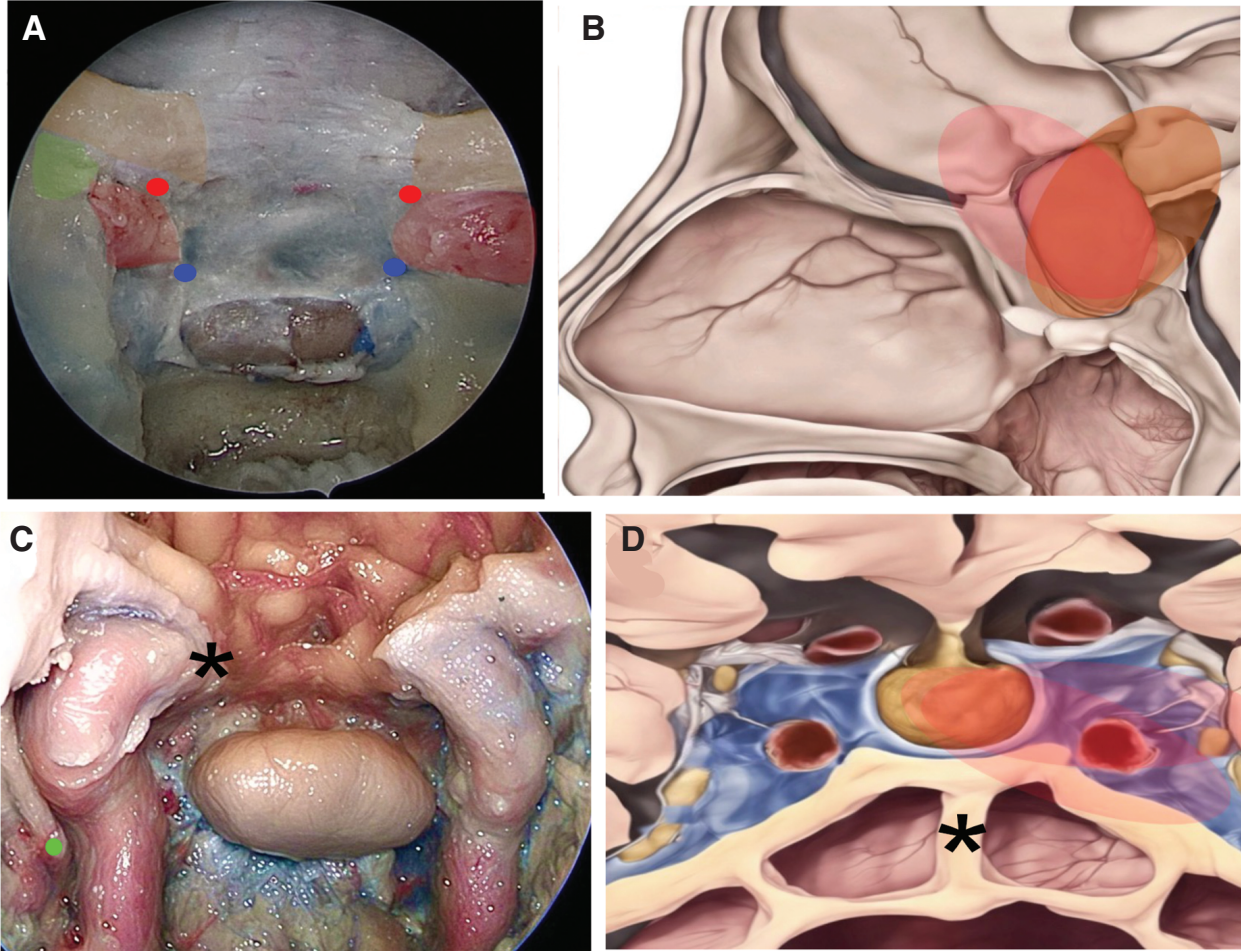
(A) Schematic illustration of the bony sellar and parasellar anatomy from an endoscopic endonasal perspective. The tuberculum recess is limited laterally by the medial opticocarotid point (MOP) superiorly (superior dots between the ICA and optic nerves) and the caroticosellar point inferiorly (inferior dots bellow the carotid arteries). The MOP demarcates the transition between the paraclinoidal ICA and the supraclinoidal ICA and corresponds to the location of the distal dural ring. (B) A schematic sagittal view demonstrating how suprasellar tumor extension can be anterior subfrontal (pink) or posterosuperior extending into the interpeduncular fossa (dark orange). (C) Anatomic dissection of an EEA with exposure of the cavernous carotid arteries bilaterally, the optic nerves and chiasm superiorly, and the bilateral sixth cranial nerves (dot). The distal dural ring (*) on the right side corresponds to the MOP. (D) Schematic illustration showing a coronal view of the sellar and parasellar region. The pattern of lateral growth into the cavernous sinus can be seen on the left side (pale red). The sphenoid sinus and intersinus septum (asterisk) are visualized. The intersinus septum is drilled flush with the sella but care should be taken as it most commonly deviates toward one of the ICAs rather than being entirely midline as it is depicted here. Reprinted/adapted with permission from Ref.^18^ 2024, Erion Junior de Andrade, MD, MSc.

The combination of tumor biology and dural anatomy determines the force vectors acting on the sella and therefore the direction of tumor growth and invasion.^17,23,24^ Superior growth occurs when the tumor extends above the level of the tuberculum sellae, and such growth may be anteriorly oriented or posteriorly into the suprasellar cistern (Figure 1B). Depending on the extent of growth in this direction, compression of the optic chiasm can occur.^24^ Inferior growth occurs when the tumor erodes through the sellar floor and enters the sphenoid sinus^24^ Anterior growth occurs when tumor extends over the planum sphenoidale, and this may even extend beneath the inferior surface of the frontal lobes.^25^ When posterior growth occurs, the tumor can extend into the clivus, the interpeduncular/prepontine cisterns, and even compress the brainstem. Lateral growth is defined by extension into the CS^20^ (Figure 1D).

## Expanded Endoscopic Endonasal Approaches

The standard EEA for PTs has been extensively described, but the main points of the nasal and sphenoidal steps in our practice will be briefly discussed. We routinely use neuronavigation as a confirmatory tool for intraoperative anatomical localization. Lateralization of the inferior and middle turbinates is performed for exposure. The middle turbinates are preserved in most cases without limitation to the extent of exposure, as they can protect from the rare complication of nasogastric tube misplacement.^26^ For PTs, we reserve middle turbinectomy for transcavernous approaches, in which a significantly more lateral exposure is required.^27^ A posterior septectomy is performed to facilitate bimanual surgery, and bilateral posterior ethmoidectomies are performed to provide adequate visualization and space. The sphenoid ostia are exposed through the resection of bilateral superior turbinates. The sphenoid ostia are cannulated and expanded with drilling of the sphenoid rostrum to complete a wide sphenoidotomy.

Upfront harvest of a nasal septal flap (NSF) is not commonly performed in our practice for PTs, even when there is an extension beyond the sella turcica. Instead, a rescue flap is fashioned which can be converted to an NSF for repair when a high-flow CSF leak is present with a large skull base defect^28^ We commonly use a semi-rigid dural substitute for epidural inlay coverage in conjunction with a commercially available dural sealant for repair. By only selectively harvesting an NSF, short-term nasal morbidity of crusting and obstruction can be avoided.^29^

Following exposure of the sellar prominence and removal of the sphenoidal mucosa, drilling of the bone overlying the sella turcica is performed and expanded with Kerrison rongeurs to the bilateral cavernous ICA’s laterally, the sellar floor inferiorly and the tuberculum superiorly.

### Transtuberculum–Transplanum Approach

**Figure 2.**
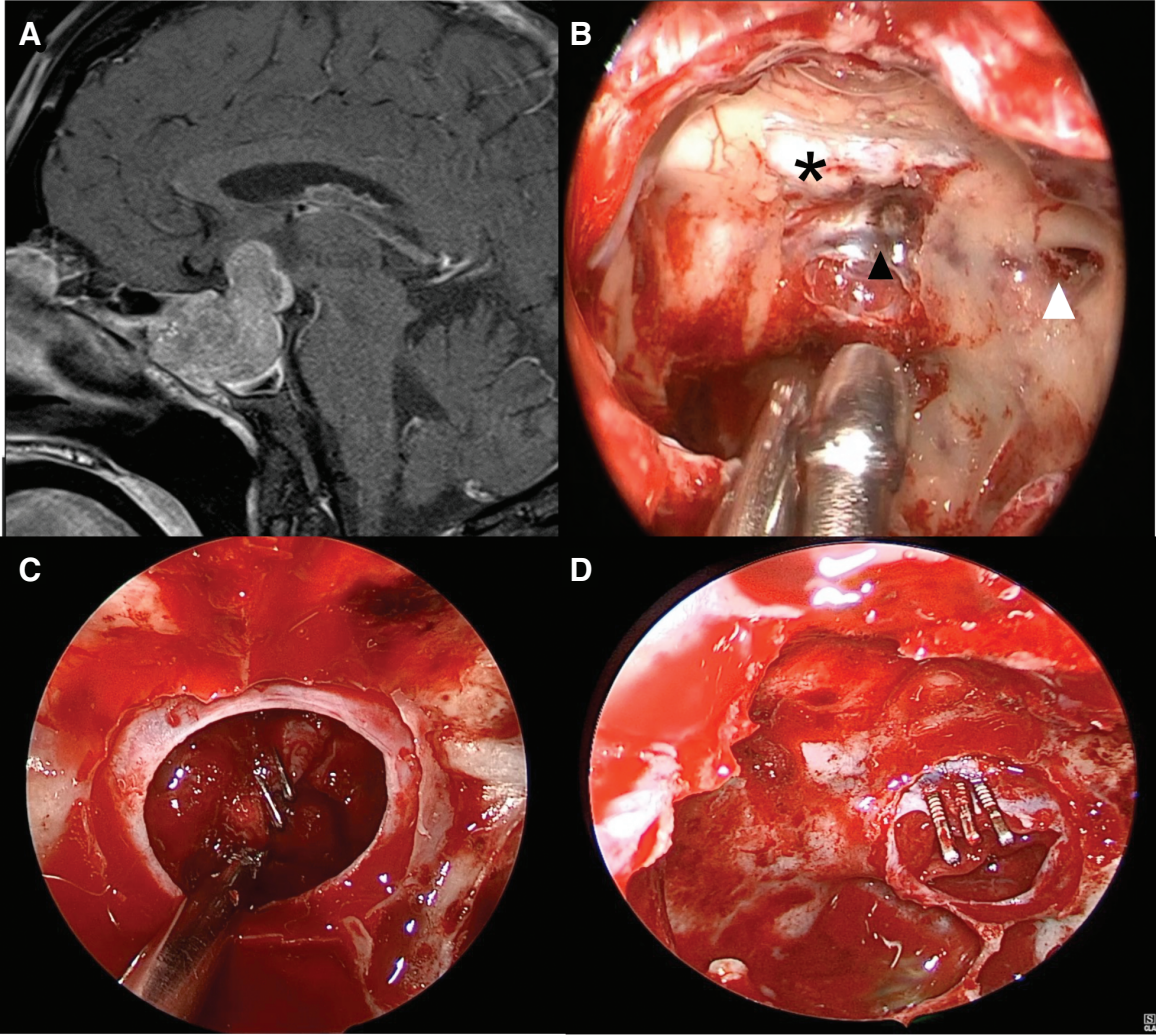
(A) Sagittal T1 with gadolinium contrast-enhanced MRI demonstrating a pituitary macroadenoma with extension into the sphenoid sinus and above the planum sphenoidale. The tumor has a dumbbell configuration indicating extension through the diaphragma sellae. To achieve suprasellar tumor resection requires a transtuberculum–transplanum (TT-TP) approach. A CSF leak should be anticipated in such cases. (B) Anatomical dissection of an EEA after a TT-TP approach. The bone of the tuberculum and planum has been resected indicated by the asterisk. The lateral opticocarotid recess (white arrowhead) corresponds to the base of the optic strut and medial to this lies the clinoidal segment of the internal carotid artery. Prior to opening the dura of the tuberculum, the superior intercavernous sinus (black arrowhead) should be cauterized and cut. (C) Weck clip repair of CSF leak from a defect in the diaphragma sellae. By reducing or resolving a CSF leak in this manner can reduce the risk of postoperative CSF leak, turning a high-flow CSF leak to either a low-flow leak or resolving the leak, which impacts subsequent reconstruction. (D) Weck clip repair of an anterior–superior CSF leak. CSF leaks at this site can be difficult to treat. One approach is to tamponade the leak by buttressing the gland to the dura with Weck clips as shown.

The TT-TP approach is an extension of the endoscopic approach in the sagittal plane (Figure 2). Anatomically, the tuberculum recess appears within the sphenoid sinus as a horizontal depression between the sellar prominence inferiorly and the planum superiorly. The recess marks both the tuberculum sellae, which is located at the level of the recess or within 2.5 mm of it as well as the diaphragma sellae.^30^ The lateral limits of the recess are the medial opticocarotid point (MOP) superiorly and the caroticosellar point inferiorly^30^ (Figure 1A). An important distinction is that of the MOP which demarcates the transition of the paraclinoidal ICA to the supraclinoidal ICA (distal dural ring) (Figure 1C) and the middle clinoidal process (MCP) which lies inferior and medial and signifies the transition between the cavernous and paraclinoidal ICA.^31^ Superior to the tuberculum sellae, the chiasmatic recess and limbus sphenoidale lead to the planum sphenoidale, which represents the horizontal superior surface of the sphenoid body.

In the context of PTs, the TT-TP approach is necessary for around 2%–3% of cases.^32,33^ Key indications include tumors that arise near the infundibulum or macroadenomas with suprasellar/supradiaphragmatic or subfrontal extension.^32^ Of these, those with suprasellar/supradiaphragmatic extension are the most common indication and these can be subclassified according to the level of their superior extent; to the suprasellar cistern, third ventricle or foramina of Monro.^33^

After the nasal phase and sphenoidal phase previously discussed, the TT-TP approach starts with assessing with neuronavigation the extent of planum exposure required to access the anterosuperior extent of the tumor. The planum sphenoidale is then drilled accordingly using the drill in a posterior-to-anterior motion. By drilling the sellar face and the planum sphenoidale first, exposure of dura above and below the tuberculum sellae provides a gauge of depth that facilitates safer drilling of the tuberculum sellae and chiasmatic sulcus. Drilling of the tuberculum sellae is performed using the drill in a superior to inferior motion while respecting the sellar and planar dura.

The sellar dura is then opened with a sickle knife and the tumor is resected inferiorly along the sellar floor and then laterally along the medial CS walls prior to resecting the tumor located superiorly. This prevents early CSF leak and helps identify the normal gland for preservation. In our experience, resection of the intrasellar component of the tumor first can facilitate suprasellar tumor descent and therefore reduce the extent of superior dural opening required. Once this is complete, the dura at the level of the superior intercavernous sinus is cauterized and cut (Figure 2B), and the dural opening is extended to the level of the superior margin of the tumor. Care should be taken on the lateral extent of the dural opening in the region of the MOP/distal dural ring as the ophthalmic artery origin is usually within 3 mm of this landmark.^34^

A potential risk of intrasellar tumor resection prior to suprasellar tumor resection is that of “intraoperative apoplexy” whereby venous congestion occurs in the suprasellar tumor due to preserved arterial inflow but the venous outflow has been eliminated by resection of the sellar tumor in proximity to the medial CS wall.^35^ It has been described that the resultant venous congestion causes a change in consistency and adherence of the residual suprasellar tumor to adjacent structures, therefore, rendering it more difficult to resect. Pezutti et al. have, therefore, proposed their “second floor strategy” whereby the dura of the planum is opened first, and the suprasellar component of the tumor within the subarachnoid space is resected.^35^ This is followed by resection of tumor within the sella and lastly by cauterization of the superior intercavernous sinus, opening of this dura along with the diaphragma sellae, and resection of the intervening tumor.

Commonly cited limitations of the endoscopic TT-TP approach are tumor extension lateral to the optic canal, vascular encasement, and unfavorable tumor consistency.^36^ With the availability of a multitude of endoscopic instruments that facilitate microsurgical dissection as well as ultrasonic aspirators and side-cutting aspiration devices, we believe that the only true limitation to the approach is tumor extension lateral to the optic canal. Di Somma et al., in their cadaveric study, have described the removal of the lamina papyracea and drilling of the medial portion of the lesser sphenoid wing and anterior clinoid process to reach the roof of the optic canal and intradurally, as far as the MCA bifurcation and insula.^37^ Our only caution against such a wide and irregularly shaped opening is the ability to achieve an effective reconstruction. Removal of the lamina papyracea can create difficulty in achieving adequate apposition of an NSF to bone and vastly increase the likelihood of reconstructive failure and frontal lobe herniation.

Complications of the approach are like those of the standard transsellar approach and include meningitis, cranial nerve or vascular injury, pituitary dysfunction, and CSF leak, although the occurrence of postoperative CSF leak is regarded to be higher given that diaphragmatic breach and resultant CSF leak are necessary to resect tumors with suprasellar extension.^32,33,38^ In such cases, vascular weck clips can be utilized to either reapproximate diaphragmatic defects or approximate the gland to the dural edge creating a physiological plug^39^ (Figure 2C and D). This can convert a high-flow CSF leak scenario to either a low-flow leak or no leak. As such, an NSF is reserved for cases in which leak repair was not possible by this means and a persistent high-flow leak remained. When a low-flow leak or no-leak scenario is encountered, it is managed according to our reconstructive ladder.^40^ The ability to manage CSF leaks in this manner facilitates the prioritization of aggressive tumor resection, particularly in cases of functional tumors.

### Transclival Approach

**Figure 3.**
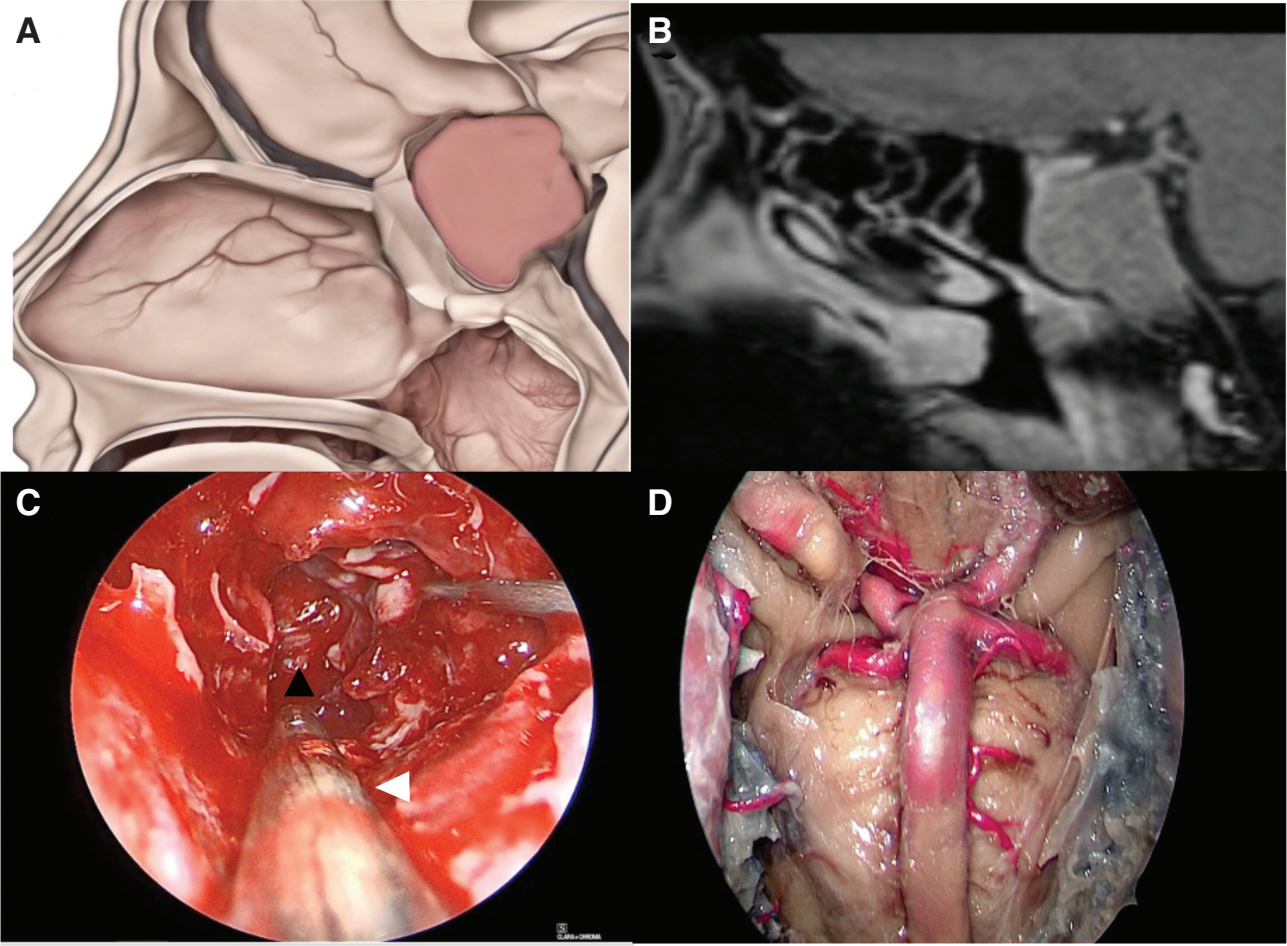
(A) Schematic illustration from a sagittal view demonstrating a pituitary macroadenoma with suprasellar and posterior fossa extension having eroded through into the clivus. (B) Sagittal T1 contrast-enhanced MRI of a pituitary macroadenoma extending anteriorly into the sphenoid sinus and inferiorly into the clivus. (C) Intraoperative image of a transclival EEA for pituitary macroadenoma resection. To reach the inferior most extension of the adenoma requires drilling of the anterior face of the sphenoid sinus down to its floor (white arrowhead). As the tumor was debulked, it was apparent that the transclival tumor extension was epidural and the intact dura of the posterior fossa could be seen (black arrowhead). (D) Anatomical dissection of a transclival EEA and dural opening exposing the interpeduncular fossa.

The clivus is a bony structure formed by the sphenoid and occipital bones, and its superior third represents the inferior and posterior osseous margin of the sella turcica. When analyzing neuroimaging of patients with large PTs, it is noted that remodeling of the dorsum sellae is a common finding, although clival bone marrow replacement by tumor or penetration through the clival dura into the posterior fossa is unusual^25,41^ (Figure 3).

PTs can invade the cancellous bone of the clivus but are usually restricted by the stronger cortical bone. When they do infiltrate the cancellous bone, they do so within the spaces of the honeycomb architecture, preserving the bony trabeculations. This makes the surgical resection of the tumor within the bone more challenging.^17,25^

PTs usually do not extend below the superior two-thirds of the clivus and can therefore be readily approached through a transsphenoidal endonasal route. In most cases, PTs with clival invasion do not breach the epidural space, and given that the sella is usually expanded by the tumor, minimal drilling of the clivus is required^25,42^ Traditional approaches to the upper clivus and interpeduncular fossa describe pituitary transposition as a necessary step, but this is not needed as the large tumor and sella expansion provide an adequate surgical corridor (Figure 3C). In cases of posterior fossa dural invasion, the bony corridor should be carefully studied to ensure that the entire dural defect is exposed. If this is not the case, additional bony resection may be required to improve the working corridor for maximum safe tumor resection. After sufficient exposure, the dural defect is visualized and can be widened if necessary. The cisternal portion of the abducens nerves can be identified bilaterally as they enter Dorello’s canal. Care should be taken in the interpeduncular space to identify and preserve the oculomotor nerves and critical vasculature.^42–44^

### Transcavernous Approach

**Figure 4.**
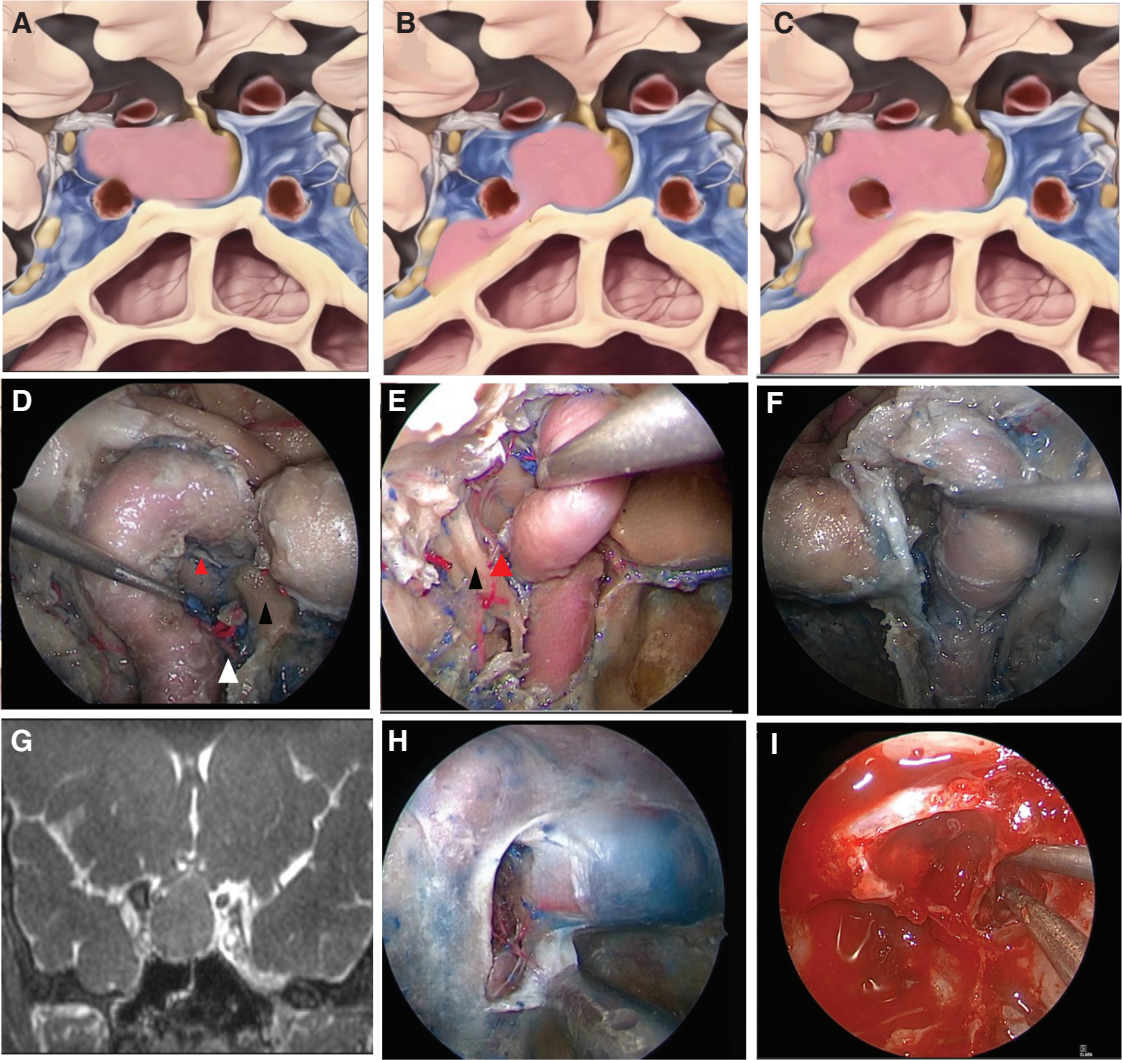
(A–C) Schematic illustration from a coronal perspective demonstrating a pituitary macroadenoma invading the superior compartment of the cavernous sinus classified as Knosp 3A (A), the inferior compartment or Knosp 3B (B) and complete invasion encasing the ICA which is Knosp 4 (C). (D) A 45° endoscopic view of an anatomical dissection of the superior compartment of the right cavernous sinus (CS). The inferior hypophyseal artery (IHA; white arrowhead), posterior clinoid process (black arrowhead), and interclinoid ligament (lateral arrowhead) can be seen. (E) A 30° endoscopic view of anterior compartment of the right CS. After transection of the proximal dural ring, the ICA can be mobilized, exposing the cranial nerves running in the lateral wall of the CS. The meningo-hypophyseal trunk (medial arrowhead) can be seen branching from the cavernous ICA and is the origin of the IHA. Its proximity to CN VI (black arrowhead) can be seen. (F) An endoscopic view of an anatomical dissection of the medial wall of the left cavernous sinus (MWCS). After opening of the anterior wall of the CS and lateralization of the ICA, the medial wall can be isolated. (G) Coronal CISS sequence of a pituitary tumor. The high resolution of this MR sequence enables us to see CS invasion on the right side but the absence of invasion on the left. In this patient with gigantism, the MWCS was resected. (H) An endoscopic anatomical dissection demonstrating the opening of the anterior wall of the CS and exposure of the parasellar ligaments. The anterior wall of the CS consists of a single dural layer whereas the dura anterior to the sella is double layered. (I) Intraoperative endoscopic view of the MWCS. Following tumor resection within the sella, an opening was made on the anterior wall of the CS. The IHA was cauterized and cut and the parasellar ligaments were incised to dissect the wall free from its attachments prior to its resection.

CS involvement is a critical independent predictor of unfavorable hormonal outcomes in functioning PTs^45^ (Figure 4). Residual tumor in the CS can result in failure to achieve biochemical remission or lead to recurrence. An important distinction is that of medial wall involvement which describes tumor adherent to the medial wall of the CS (MWCS) and CS invasion which describes tumor breach through the medial wall into the CS.^46^ Careful evaluation of preoperative 3-dimensional constructive interference in steady state (3D CISS) or fast imaging employing steady-state acquisition-cycled phases (FIESTA-C) magnetic resonance sequences to assess the integrity of the medial wall can sometimes inform the surgeon as to whether true invasion is present^47^ (Figure 4G).

Several anatomical classifications of the CS have been proposed. Harris and Rhoton categorized the CS into 4 venous spaces based on their relationship with the internal carotid artery (ICA): anteroinferior (ventral), posterosuperior (dorsal), medial, and lateral.^48^ In 2017, Fernandez-Miranda et al. described 4 compartments in relation to the cavernous ICA: superior, posterior, lateral, and inferior.^49,50^ In a recent study, recognizing that some of those compartments are divided only by virtual lines, we have described the division of the CS into 2 main compartments; posterosuperior describing the compartment posterior and superior to the ascending and horizontal segments of the cavernous ICA respectively (Figure 4D) and anterior describing the compartment anterior and inferior to the ascending and horizontal segments of the cavernous ICA^19^ (Figure 4E). We believe that this classification provides clinical utility when deciding on the best surgical approach to tumors in these regions.

The extent of CS invasion by PTs has been described in a variety of ways.^4,5,47,51,52^ The most widely utilized classification was published by Knosp et al. where tumor invasion is categorized into 6 grades (0, 1, 2, 3a, 3b, 4) based on the relation to 3 lines drawn between the supraclinoid ICA and the horizontal segment of the cavernous ICA on coronal MR imaging^5,47^ These lines are described as the medial tangent, intercarotid line, and lateral tangent. Knosp grades 3 and 4 can be defined as invasive to the CS (Figure 4A–C).^47,53^ Interestingly, grade 3a tumors which extend superiorly into the CS have been shown to more commonly displace the MWCS whereas grade 3b and 4 tumors which extend into the CS inferiorly are more likely to represent true invasion of the CS^54^ (Figure 4).

#### EEA to the anterior space of the cavernous sinus.—

The anterior space of the CS can be further subdivided into an inferior and lateral compartment by a line extending perpendicular to the horizontal portion of the cavernous ICA.^19^ The lateral compartment contains the cranial nerves of the CS and posteriorly relates to the geniculate ganglion and Meckel’s Cave.

When approaching the anterior space of the CS endoscopically resection of the ipsilateral middle turbinate and drilling of the pterygoid base to expose the vidian nerve facilitates lateral exposure.^19^ If there is significant tumor extension into the lateral compartment, the vidian nerve may need to be sacrificed to achieve additional exposure and maneuverability. The anterior wall of the CS is then exposed via drilling of the bone directly overlying the cavernous and clinoidal ICA, starting inferiorly at the level of the sellar floor and migrating superiorly.

Once the anterior wall of the CS is exposed, a microdoppler is used to confirm the location of the ICA and the periosteal layer of dura between the gland medially and the ICA laterally is incised (Figure 4H). The cut is initiated inferiorly where there is the greatest separation between the 2 structures.^43^ A hemostatic agent consisting of gelatin matrix and human-derived thrombin is injected into the CS opening and a cottonoid is placed to achieve hemostasis.

If there is an extensive tumor present lateral to the ICA, the proximal dural ring can also be cut to mobilize the ICA and provide space to explore between the artery and the cranial nerves (Figure 4E). Care should be taken to preserve the ipsilateral abducens nerve which is lateral to the ICA and follows a medial trajectory in relation to the ophthalmic segment of the trigeminal nerve^19,49^ (Figure 1C).

#### EEA to the posterosuperior space of the CS.—

The posterosuperior cavernous space is located posterior to the paraclival ICA and superior to the horizontal segment of the cavernous ICA.^19^ It can be subdivided into posterior and superior compartments, respectively.^49,55^ The superior compartment correlates with the oculomotor triangle when approached intracranially and is in contact with the posterior compartment of the CS at the posterior genu of the cavernous ICA. The boundaries of the superior compartment are the roof of the CS superiorly, the lateral wall of the CS laterally, the MWCS medially, and the horizontal segment of the cavernous ICA inferiorly.^19^ The posterior clinoid process, the interclinoidal ligament, and CN III can all be found in the superior compartment (Figure 4D). Access to the posterosuperior cavernous space is through the MWCS. Tumor within the sella is resected first to expose the medial wall and once this is opened, the superior compartment can be visualized superior to the horizontal segment of the ICA.^19^ A prominent medial clinoid process (MCP) can obstruct access to the superior compartment and as such should be drilled and resected from lateral to medial.^19^ Access to the posterior compartment requires identification of the posterior genu and ascending segment of the carotid.^19,50^ Care should be taken when dissecting toward the posterior genu to identify the inferior hypophyseal artery which arises from the meningo-hypophyseal trunk and has a lateral to medial trajectory (Figure 4D). It is important to coagulate and cut this vessel to prevent avulsion from the ICA. Angled endoscopes can be used to visualize the space posterior to the paraclival ICA where the medial petrous apex, meningo-hypophyseal trunk, and abducens nerve can be seen.^19^ CN VI enters the CS through Dorello’s canal and runs in posterolaterally to the ascending cavernous ICA following which it travels inferior to the horizontal segment and towards the superior orbital fissure.

#### Endoscopic endonasal resection of the medial wall of the cavernous sinus.—

The medial wall of the cavernous sinus (MWCS) is the lateral limit of the sellar space and is a frequent site of PT invasion. The likelihood of tumor infiltration of the wall has been shown to correlate with increasing Knosp grade and has been identified as a significant factor linked to persistent hormonal disease and tumor recurrence in PTs.^52,56^ Mohyeldin et al. have also demonstrated that different subtypes of PTs have a varying predilection for medial wall infiltration with somatotrophic tumors having the highest frequencies of involvement at each Knosp grade.^52^ Furthermore, the authors reported that with resection of the medial wall, remission rates of 92% were achieved in their retrospective series as compared to rates of 55% quoted in the literature.^57^ This, however, was based on short-term follow-up. Evidence for resection of the MWCS in other PT subtypes is still lacking but may be a consideration for medically refractory prolactinomas and PT subtypes for which stereotactic radiosurgery is less effective.^52,58,59^

The surgical technique for resection of the medial wall requires exposure of the sellar dura, clinoidal ICA, and at least the superior portion of the anterior wall of the CS^19,49,60^ (Figure 4). After the transsellar approach and tumor removal, angled endoscopes can be used to inspect the medial surface of the MWCS for tumor invasion. Two main techniques of medial wall resection have been described; “medial to lateral” and “anterior to posterior”^20,60–64^ (Figure 4I). The practice of our senior author is the latter technique with the opening of the anterior wall of the CS and then an anterior-to-posterior dissection to separate the medial wall from the ICA.^52,63,64^ Prior to opening the anterior wall of the CS, doppler ultrasound is used to map the trajectory of the ICA and determine a safe entry point for opening with a McElveen knife. Once the CS is open, direct injection of an injectible hemostatic is used with gentle tamponade with a cottonoid. An anterior-to-posterior dissection technique is then used to manipulate the MWCS away from the carotid artery (Figure 4F). The dissection requires cutting of the inferior parasellar ligament following which the inferior hypophyseal artery is cauterized and cut. The dura overlying the posterior clinoid is then dissected until the carotico-clinoidal ligament is seen and cut. Lastly, a cut is made through the dural attachments to the proximal dural ring.^52^

In the medial to lateral technique, the inferior intercavernous sinus is entered and this plane is used to dissect the MWCS from the anterior wall of the CS.^20,61,62^

### Reconstruction Following Expanded EEAs

Specific factors should be taken into consideration when determining the method of skull base reconstruction. First, the presence or absence of CSF leak. If a leak is apparent, it should be classified as either low- or high-flow leak. As mentioned earlier, we are at times able to reduce and sometimes resolve an intraoperative CSF leak with vascular Weck clips, and this can positively alter the degree of endoscopic reconstruction required. Second, the size of the skull base defect—with larger defects requiring vascularized flaps and lastly, exposure of the ICA. In situations of absent CSF leak and small defects, a collagen-based (CB) dural replacement graft or free mucosal graft is sufficient.^13^ In a low-flow CSF leak, a multilayer closure with both CB dural graft and free mucosal graft are used in our practice. Lastly, for high-flow leaks, large defects, and/or exposure of the ICA, a multilayer closure with a CB dural graft (most commonly a 2-layer button graft with an inlay and onlay component) along with an NSF is preferred. The last step of all these techniques is the use of a commercially available dural sealant. A fascia lata autograft is rarely used in our practice in the context of PTs and is generally reserved for cases of high-flow CSF leak in which a sufficient NSF could not be harvested due to perforations from prior sinus surgery.^40^

The most utilized closure technique in our practice for uncomplicated PTs without CSF leak is that of a CB dural graft, for epidural or subdural coverage, followed by a dural sealant.

### Predicting the Extent of Resection

A key challenge in PTs, particularly in giant tumors, is the ability to preoperatively predict the extent of tumor resection achievable. As such, several groups have developed scoring systems to facilitate this prediction.^51,65,66^ The TRANSSPHER score allocates a point for; tumor diameter >40 mm in any plane, Knosp grades 3–4, and nodular extension into the frontal lobe, temporal lobe, posterior fossa, or ventricle.^51^ The resultant scores of 0–3 were shown and validated to inversely correlate with the likelihood of gross total resection (GTR; grade 0: 89%, grade 1: 54%, grade 2: 25%, and grade 3: 17%). These results reflect tumors operated on by endoscopy and microscopy. In our experience, tumor consistency and vascularity likely play a role but, to our knowledge, there are currently no imaging characteristics that can adequately predict this preoperatively.

Ceylan et al. published their retrospective series of patients with giant tumors who underwent only endoscopic resections.^65^ They identified several tumor characteristics that negatively impacted the extent of resection: invasion of the lateral CS, tumor extension into the anterior cranial fossa, posterior cranial fossa or inferior wall of the lateral ventricle, a multilobulated configuration, suprasellar extension exceeding 2c m in height, and the ratio of the horizontal suprasellar tumor length to the distance between the supraclinoid carotids exceeding 1.5. Unsurprisingly, these findings correspond to those of the TRANSSPHER study but with the addition of the latter 2 factors which pertain to the limits of reach with the endoscopic approach.

In instances where subtotal resection is likely, it is also not clear which surgical strategy is best—whether to perform the endoscopic approach first, the transcranial first or whether to perform a combined approach from the outset. Given the risk of apoplexy arising in the suprasellar tumor residuum, if an endoscopic approach is performed first, our preference is to select a transcranial approach that provides us with the greatest access to achieve a GTR in a single operation.

## Discussion

Advances in our understanding of parasellar anatomy and the development of high-resolution endoscopic visualization as well as a multitude of endoscopic instruments that facilitate microdissection have led to the progress in expanded EEA and the ability to achieve improved resection rates in pituitary macroadenomas. Furthermore, the initial challenges of these expanded approaches, including CSF leak and cranial nerve injury have been managed with evolving reconstructive techniques and operative adjuncts such as neuromonitoring, ultrasound microdoppler, and neuronavigation. The expanded approaches discussed in this article are, however, more technically demanding and are associated with incrementally higher complication rates even in experienced skull base centers. As such, a graded experiential approach to undertaking these cases is recommended for surgeons entering the field.^67^

Despite the advances mentioned, achieving GTR in pituitary macroadenomas continues to pose an operative challenge, and it is, therefore, important to consider the reported outcomes in the literature pertaining to these expanded approaches.

### Outcomes After Expanded EEAs

Elshazly et al. reported their outcomes in a series of 55 patients with giant PTs. GTR was achieved in 44% of patients and near-total resection (NTR; defined as >90%) was achieved in 47%. They identified CS invasion and a multilobular configuration as statistically significant factors that limited the degree of tumor resection. However, no transcavernous approaches were described in their series and 69% of patients were reported as having a preoperative Knosp grade of 3–4. An important finding of this study was that all 4 functional tumor patients did not achieve biochemical remission with surgery alone and required adjuvant medical or radiation therapy to do so. This is a key factor to discuss preoperatively with patients who have a functional PT. New-onset pituitary hormone deficiency was the most common complication with a rate of 17%.

A larger series published by Ceylan et al. reported on a series of 205 patients with giant tumors of which 49 patients underwent an expanded EEA.^65^ GTR was achieved in 35% of patients, NTR in 40%, and subtotal resection in 25%. Interestingly, the group reported on their transition to a more aggressive surgical approach after 2017 with the adoption of expanded EEA and demonstrated increased rates of GTR (40% vs. 27%) and NTR (41% vs. 38%) with a reduced complication rate. Similarly, to the prior series, new-onset hormonal deficiency was the most common postoperative complication with a rate of 22%–23% that remained stable even after the adoption of expanded approaches.

### Outcomes in Patients With CS Invasion

In a series of 137 patients, the correlation between the Knosp classification on preoperative MR imaging was correlated with intraoperative endoscopic visualization of CS invasion.^56^ Tumors classified as grade 3 radiologically had a corresponding invasion rate of 37.9% (3A: 26.5%; 3B: 70.6%) and all tumors classified as grade 4 proved to be invasive. Rates of GTR in grade 3 tumors was 64% and in grade 4 tumors was 0%. Ajlan et al. reviewed their outcomes in 176 endoscopically resected PTs, where 77% of cases were macroadenomas and CS involvement was seen in 23% of patients.^45^ Despite the adoption of both a medial and lateral transcavernous approach for resection of invading tumors, they reported that the presence CS invasion resulted in a significantly lower likelihood of achieving GTR (47% compared to 86% in tumors without CS invasion). As such, there was also a higher need for subsequent interventions (40% vs. 4%). The presence of CS invasion also resulted in lower rates of hormonal remission in functional adenomas (15% vs. 63%). The expanded transcavernous approach did not result in any additional morbidity when compared to the standard transsellar approach in this series.

### Outcomes in Patients Who Underwent MWCS Resection

In a recent meta-analysis, De Macêdo Filho et al. reported on 5 studies with a total of 208 patients. Disease remission was reported in 95% of patients with a complication rate of 4.8%. Median follow-up was between 11 and 30 months, which is relatively short in terms of PT follow-up. The most common complication was that of transient diplopia. Interestingly, the prevalence rate of CS invasion was between 10.4% and 36.7%, which may reflect the fact that 83% of tumors included in these studies were of Knosp grade 0–2.

### Risk of Postoperative Apoplexy

An important risk of endonasal surgery for PTs and particularly giant tumors is that of STR leading to apoplexy of the residual tumor. Postoperative apoplexy (PoA) is regarded as more hazardous than spontaneous apoplexy with significantly greater morbidity and higher rates of mortality.^68–70^ The latter has been attributed to hypothalamic and electrolyte dysfunction.^69^

Butterfield et al. performed a meta-analysis of 17 studies and 1031 cases of giant pituitary adenoma resection.^70^ The rate of STR was 35.6% with PoA occurring in 1.84% of patients overall and 5.65% of patients with STR. The mortality rate of patients with PoA was 42% and the most common timing of the apoplexy was within 24 hours of surgery. Given the high mortality rate and the timing of events, the authors advocated for combined approaches to be performed on the same day or on concurrent days.

It is, therefore, imperative to consider the likelihood of achieving GTR in the preoperative planning of giant pituitary macroadenoma surgery. If combined approaches are to be performed on different days, a transcranial procedure is recommended first to resect tumor within the suprasellar/intradural space where the risk of hemorrhage is associated with more significant consequences than tumor hemorrhage within the sella or CS which are regarded as interdural spaces.^70^

### Future Directions

As the field of endoscopic skull base surgery continues to evolve, ongoing research and clinical experience will further refine these approaches and extend the boundaries of what is surgically accessible. A key challenge in functioning tumors, particularly in Cushing’s disease, is the ability to localize the tumor within the sella. This is further compounded in cases of failed remission or disease recurrence. Hence enhanced preoperative imaging modalities and intraoperative visualization systems are much needed and could potentially provide the greatest benefit in achieving disease remission for patients. In addition, the ability to intraoperatively determine whether the pituitary adenoma has infiltrated the MWCS prior to attempted resection would ensure that patients are not subjected to the undue risk of possible diplopia and carotid artery injury.

Although beyond the scope of this article, there are increasing efforts to determine the molecular events leading to the formation and propagation of PTs. Hence there may someday be molecularly targeted therapies that complement surgical intervention and reduce the need for extensive resections in certain PT subtypes.

These innovative directions are anticipated to enhance the efficacy of pituitary surgery, thereby improving the prognosis and quality of life for patients with PTs.

## Conclusions

Innovations in endoscopic technology and a deeper understanding of surgical anatomy have greatly enhanced the feasibility of safely and effectively resecting invasive PTs. This has had significant clinical implications with improvements in rates of gross total tumor resection as well as enhanced disease remission rates in functional adenomas. In experienced hands, the morbidity of expanded EEAs including entering the CS is low and with reconstructive techniques, CSF leaks can be well managed. However, the complexity of these approaches underscores the necessity for meticulous planning, a comprehensive understanding of the relevant neurovascular anatomy, and a multidisciplinary skull base team.
